# Aspects épidémiologiques des fractures de membres liées à l'exercice de la fonction militaire au Togo

**DOI:** 10.11604/pamj.2015.20.377.6340

**Published:** 2015-04-16

**Authors:** Yao Messanvi Akpoto, Anani Abalo, Faré Gnandi-pio, Lantam Sonhaye, Mazamaesso Tchaou, Hamza Doles Sama, Sarakawabalo Assenouwe, Damessane Lamboni, Kossigan Adodossi Amavi, Saliou Adam, Essossinam Kpelao, Kodjo Tengue, Badjona Songne-Gnamkoulamba

**Affiliations:** 1 Service d'Orthopédie Traumatologie du CHU Sylvanus Olympio de Lomé, Togo; 2 Service d'Orthopédie Traumatologie du CHU Kara, Togo; 3 Service de Radiologie CHU Lomé, Togo; 4 Service des Urgences Chirurgicales du CHU Sylvanus Olympio de Lomé, Togo

**Keywords:** Fractures de membres, force de défense et de sécurité, formation militaire, Togo, limb fractures, security forces, military, Togo

## Abstract

Le but de notre étude était de déterminer la fréquence des fractures de membres liées à l'exercice de la fonction militaire au sein des Forces de Défense et de Sécurité en milieu africain en vue de ressortir l'impact des différentes circonstances de survenue. Nous avons entrepris une étude rétrospective descriptive allant du 1er janvier 2004 au 31 décembre 2013. Elle a concerné les agents des forces de défense et de sécurité traités pour des fractures de membres au cours de cette période. Sept cent quatre (704) cas de fractures de membres ont été dénombrés. L’âge moyen des patients était de 30,57 ans avec des extrêmes de 19 et 55 ans. La prédominance masculine était nette (95,71%). L'Armée de Terre (51,05%) et la Gendarmerie Nationale (38,86%) étaient les plus représentées. Les hommes du rang étaient majoritaires (43,08%), suivis des sous-officiers (32,59%). La fréquence annuelle des fractures de membres en rapport avec la profession militaire était de 63 cas. Les fractures de jambe étaient les lésions les plus recensées (32,96%). Les Formations et les stages militaires ont été les circonstances de survenue les plus rencontrées (42,60%), suivies des accidents de la circulation (39,43%). La perte des journées de service liée à ces lésions était estimée à 14009 jours par an. Les fractures de jambes occupent le premier rang des fractures de membres en rapport avec l'exercice de la profession militaire. Les formations-stages militaires et les accidents de la voie publique en sont les deux grandes circonstances de survenue.

## Introduction

Les lésions musculo-squelettiques constituent un problème majeur de santé publique dans les armées en raison des conséquences en terme de morbidité, de perte de main-d´œuvre, de diminution de la capacité opérationnelle des troupes et des énormes coûts liés à leur prise en charge [1, 2]. Bien que la pathologie traumatique des membres constitue un fléau qui touche toutes les couches sociales, il faut souligner que les agents des Forces de Défense et de Sécurité (FDDS) constituent une population hautement exposée. En dehors des étiologies habituelles des fractures de membres, certaines activités comme les formations et les stages militaires sont reconnues comme particulièrement à risque, tant sur le plan de la fréquence que de la gravité des accidents. Selon Tomlinson et al. [3] ces lésions musculo-squelettiques représentaient 53% de toutes les blessures liées à l'entrainement et à l'exercice physique surtout dans les unités de combat. Des séries américaines et européennes [1, 2, 4] ont étudié les différents aspects de ces fractures de membres dans les armées. Nous avons donc entrepris ce travail dont l'objectif était d’étudier les aspects épidémiologiques et lésionnels de ces fractures, en vue de ressortir l'impact des différentes circonstances de survenue en milieu africain.

## Méthodes

Il s'est agi d'une étude rétrospective descriptive sur une période de dix ans (du 1er janvier 2004 au 31 décembre 2013). Elle a porté sur les dossiers de 629 agents des Forces de Défense et de Sécurité (FDDS). Etaient inclus dans cette étude les patients dont la fracture était survenue pendant une formation ou un stage militaire, pendant un sport lié au service militaire, ou au cours de l'exécution d'une mission ordonnée par l'armée. Etaient exclus les patients dont les dossiers étaient incomplets et les patients décédés ou admis à la retraite avant l'enquête. Les paramètres suivants ont été étudiés: l’âge, le sexe, le grade du patient, l'armée d'incorporation, les circonstances de survenue, les lésions présentées et la durée d'arrêt de travail après l'accident. Nous définissons par agent des Forces De Défense (FDD) les militaires de l'armée de terre, de la marine nationale, de l'armée de l'air et de la gendarmerie nationale; et par agents des Forces De Sécurité (FDS) les fonctionnaires de la police nationale, les sapeurs-pompiers militaires et les agents de douane. Les FDD étaient composées d'officiers, d’élèves officiers, de sous-officiers et de militaires du rang. Les données recueillies sur une fiche de renseignement individuelle ont été saisies à l'aide du logiciel Epi data, puis analysées par le logiciel SPSS 10,0. Les résultats ont été considérés comme statistiquement significatifs avec la valeur de p≤ 0,05.

## Résultats

**Aspects épidémiologiques:** Pendant notre période d’étude 1366 agents des FDDS avaient présenté une fracture de membre dont 629 (46,05%) étaient survenues au cours d'une activité militaire. Il y avait 104 (16,53%) agents des FDS et 525 (83,47%) agents des FDD. La série ainsi constituée comportait 26 (4,13%) officiers, 23 (3,66%) élèves officiers, 205 (32,59%) sous-officiers et 271 (43,08%) militaires du rang (Tableau 1). La répartition des agents des FDD par rapport aux différentes armées avait permis de retrouver 268 (51,05%) militaires de l'armée de terre, 204 gendarmes (38,86%), 31 (5,9%) militaires de l'Armée de l'Air et 22 (4,19%) militaires de la Marine nationale. La fréquence annuelle des fractures de membre en rapport avec la profession militaire était de 63 cas. L’échantillon étaient composé de 602 patients de sexe masculin (95,71%) et 27 de sexe féminin (4,29%) avec une sex-ratio de 22,3. L’âge moyen global des patients était de 30,57 ± 13 ans. Les extrêmes étaient de 19 et 55 ans (Tableau 2).

**Tableau 1:** Répartition des patients selon les différentes classes au sein des FDDS

	Officiers	Elè. Off	S/Off	H/Rang	Fn. Pol	S-P. Mil	A. Doua	Total	
	n	n	n	n	n	n	n	n	%
2004	2	1	17	22	4	3	4	53	8,43
2005	4	4	23	41	5	5	4	86	13,67
2006	3	5	21	24	3	2	-	58	9,22
2007	2	-	25	31	2	4	3	67	10,65
2008	3	4	11	26	3	2	-	49	7,79
2009	1	-	20	19	5	-	3	48	7,63
2010	4	3	32	30	8	6	7	90	14,31
2011	1	2	18	18	1	3	1	44	7
2012	5	4	20	39	7	7	3	85	13,51
2013	1	-	18	21	4	3	2	49	7,79
Total (%)	26(4,13)	23(3,66)	205 (32,59)	271 (43,08)	42 (6,68)	35 (5,57)	27 (4,29)	629	100

Elè. Off: élèves officiers; S/Off: sous–officiers; H/Rang: hommes du rang; Fn. Pol: fonctionnaire de police; S-P.Mil: sapeurs-pompiers militaires; A/Doua: agents de douane

**Tableau 2:** Répartition des patients par tranche d’âge

	Hommes	Femmes	Total	Pourcentage
[19-28]	287	18	305	48,49
[29-38]	211	6	217	34,5
[39-48]	88	3	91	14,47
[49-58]	16	0	16	2,54
Total	602	27	629	100

**Les circonstances de survenue:** Les formations et les stages militaires étaient au premier rang des circonstances de survenue et étaient retrouvés chez 268 patients (42,60%). Deux cent quarante-huit patients (39,43%) étaient victimes d'accidents de la voie publique, 90 patients (14,31%) avaient eu un accident de sport et 23 patients (3,66%) étaient victimes d'accidents par arme à feu. Les formations militaires incriminées étaient la Formation Elémentaire Toute Arme (FETTA) chez 122 patients (45,52%), les stages d'avancement chez 68 patients (25,37%), le brevet de parachutisme dans 28 cas (10,45%), les arts martiaux dans 26 cas (9,70%) et la formation commando chez 24 patients (8,96%). Parmi les patients victimes d'accident de sports, 35 (38,89%) avaient reçu un choc au cours du ballon militaire, 30 (33,33%) au cours d'un match de football, 21 (23,33%) lors d'un match de volleyball et 4 (4,45%) lors d'une course à pied.

**Figure 1:**
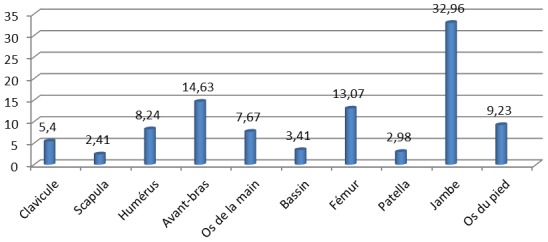
Des lésions osseuses selon leur fréquence

**Figure 2:**
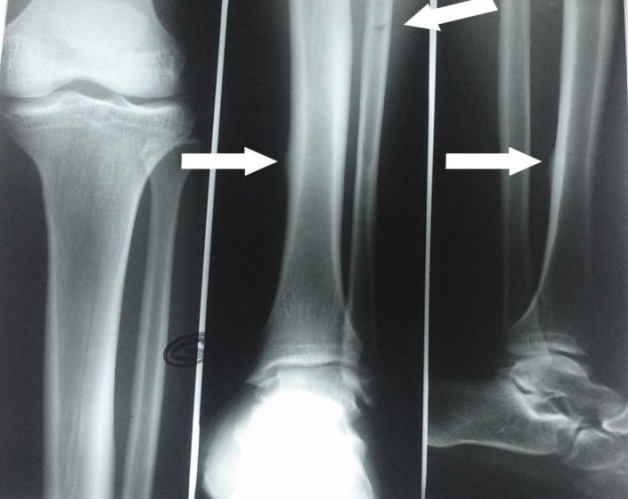
Caporal de 24 ans présentant une douleur du tiers inférieur de la jambe gauche au cours d'une marche pendant un stage militaire: radiographie de la jambe gauche de face et de profil montrant une fracture corticale pure médiale du tiers inférieur de la diaphyse tibiale

**Figure 3:**
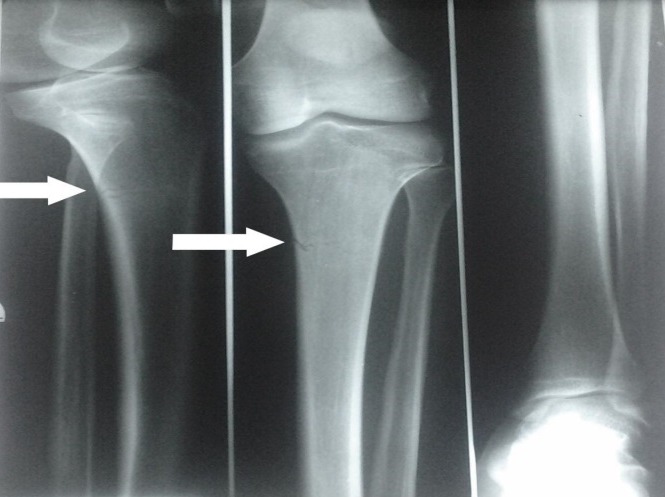
Jeune recrue de 20 ans présentant une douleur du tiers supérieur de la jambe gauche après une marche de 25 Km: radiographie de la jambe gauche de face et de profil montrant une fracture mixte corticale et spongieuse du tiers supérieur de la diaphyse tibiale

**Aspects lésionnels:** Dans l’échantillon étudié, 571 patients (90,78%) avaient présenté une fracture unique, 41 (6,51%) avaient eu une double fracture et 17 (2,7%) une triple fracture; soit un total de 704 fractures. Le membre supérieur a été atteint dans 270 cas (38,35%) et le membre inférieur dans 434 cas (61,65%). Les lésions osseuses les plus fréquentes étaient celles de la jambe dans 232 cas (32,96%). Elles étaient suivies des fractures des deux os de l'avant-bras retrouvées dans 103 cas (14,63%) puis des fractures du fémur (Figure 1). Les accidents de la voie publique étaient les seuls à occasionner les fractures doubles et triples; aussi ils sont responsables de 323 cas de fractures (45,88%). Le Tableau 3 nous donne la répartition des sièges des fractures selon les circonstances de survenue. Les fractures survenues au cours des formations militaires représentaient 38,07% de l'ensemble des lésions recensées avec 268 cas. Ces dernières ont concerné le membre supérieur dans 64 cas (23,88%) et le membre inférieur dans 204 cas (76,12%). Au niveau du membre supérieur, 25 cas (39,06%) de fractures ont concerné les os de la main dont 12 (48%) fractures de phalanges, 9 (36%) fractures des os du métacarpe et 4 (16%) fractures des os du carpe. Les fractures des deux os de l'avant-bras venaient au deuxième rang de ces lésions avec 18 cas (28,13%). Les fractures des deux os de la jambe étaient les plus recensées au membre inférieur avec 152 cas (56,72%). Elles étaient dominées par les fractures de stress qui étaient dénombrées chez 129 patients (84,87%). L'incidence annuelle de ces fractures de stress était de 3,06% (Figure 2 et Figure 3). Avec respectivement 12,78% et 3,27% de lésions osseuses, les fractures causées par les sports collectifs et les armes à feu étaient les moins recensées (Tableau 3).

**Tableau 3:** Répartition des sièges des fractures selon les circonstances de survenue

	Form. /Stage	Sports Col.	AVP	Armes à feu	Total	
					n	%
Clavicule	9	13	16	-	38	5,4
Scapula	-	-	17	-	17	2,41
Humérus	12	11	31	4	58	8,24
Os de l'avant-bras	18	17	62	6	103	14,63
Os de la main	25	10	16	3	54	7,67
Bassin	4	3	17	-	24	3,41
Fémur	11	-	77	4	92	13,07
Patella	6	4	11	-	21	2,98
Os de la jambe	152	16	58	6	232	32,96
Os du pied	31	16	18	-	65	9,23
Total (%)	268 (38,07)	90 (12,78)	323 (45,88)	23 (3,27)	704	100

Form /Stage: formations / Stages; Sports Col: sports collectifs; AVP: accidents de la voie publique

**Figure 4:**
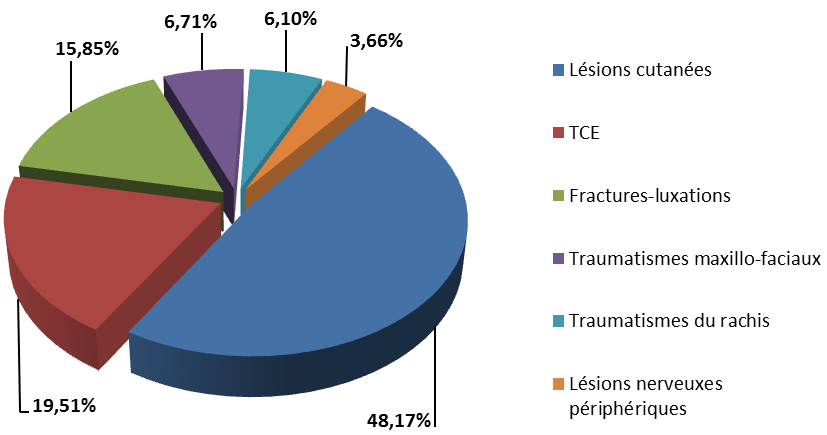
Répartition des lésions associées selon leur fréquence.

**Lésions associées:** Cent soixante-quatre patients (26,07%) avaient présenté des lésions associées (Figure 4). Il s'agissait des patients qui avaient présenté des fractures dues aux armes à feu et 141 patients (56,85%) accidentés de la voie publique. Nous n'avons pas enregistré de lésions associées aux fractures survenues lors des sports et des formations militaires. Les fractures ouvertes ont été recensées chez 79 patients (48,17%). Trente et un patients (39,24%) avaient présenté une fracture ouverte classée Gustilo I. Les lésions de type Gustilo II et Gustilo III étaient retrouvées chacune dans 24 cas (30,38%). Dans 8 cas (34,78%) de fractures dues aux armes à feu il n'y avait pas eu d'orifice de sortie; l'ouverture d'entrée a été classée Gustilo II. Pour les autres cas, l'ouverture de sortie a été classée Gustilo IIIa chez 11 patients (47,83%) et Gustilo IIIb chez 4 patients (17,39%). Les traumatismes crânio-encépahliques étaient composés de 17 cas (53,12%) de commotions cérébrales, 9 cas (28,3%) de contusions cérébrales et 6 cas (18,75%) de lésions hémorragiques intracrâniennes. Les atteintes nerveuses périphériques étaient représentées par 6 cas d'atteinte motrice du nerf radial (3,66%). Huit cas (30,77%) de fractures-luxations ont été enregistrés au niveau de l’épaule, 6 au niveau du poignet et de la cheville (23,08%), 4 cas au niveau du coude (15,38%) et 2 cas ont été notés au bassin (7,69%).

**Evaluation des pertes de journées de service:** Dans le groupe des patients ayant présenté la fracture d'un seul os, l'estimation des jours de service perdus au cours des 10 ans était de 122184 jours avec une moyenne de 214 jours de service perdu par chaque patient. Chez les patients ayant présenté une double et une triple fracture le nombre total de jours de services perdus était respectivement de 11808 et de 6103 jours; soit un total de 140095 jours perdus par tous les patients pendant les 10 ans. Les moyennes de jours de service perdus par un patient ayant présenté une double et une triple fracture étaient respectivement de 288 et de 359 jours (P≤ 0,011). Chez les patients ayant présenté des lésions associées, cette moyenne était de 311 jours (P≤ 0,038).

## Discussion

Notre travail a porté sur 629 patients sur une période de dix ans. Cette taille de notre échantillon est relativement réduite par rapport aux séries de Lauder TD. et al [5] et de Smith GS et al [6] aux USA qui ont étudié respectivement une population de 13861 et de 17718 patients. Cette différence avec notre série pourrait être liée à l’échantillonnage car ces études ont porté sur toutes les pathologies ostéoarticulaires pouvant survenir pendant les formations militaires. De plus les armées étudiées n'ont pas les mêmes effectifs et les mêmes missions. La fréquence des traumatismes de membres est élevée dans les armées. Selon Knapik JJ et al [7] ces traumatismes occupent 61% de toutes les blessures liées au service militaire. Pour Hauret KG et al [8] le ratio est de 628 traumatismes de membres pour 1000 personnes par année. Dans notre série la fréquence annuelle étaient de 63 fractures de membre par année. Dans les armées, ces traumatismes de membres touchent préférentiellement les sujets de sexe masculin. Ceci a été noté dans la série de Lauder et al [5] qui a retrouvé que 94% des hospitalisations pour des lésions dues aux sports et aux exercices physiques au sein de l'armée américaine concernaient les militaires de sexe masculin. Dans notre étude les sujets de sexe masculin étaient atteints dans 95,71% des cas. De plus ces fractures sont l'apanage des sujets jeunes et de grade inférieur. Pour Lauder et al [5], 41% de blessés étaient âgés de 20 à 24 ans avec une atteinte des sujets de grades inférieurs dans 79% des cas. Dans notre série, les patients jeunes de 19 à 28 ans représentaient 48,49% de l’échantillon avec 75,67% de sujets de grades inférieurs. Bien qu´il n´y ait pas de données au sujet de cette répartition des lésions, on peut supposer qu'elle est liée au fait que les jeunes soldats, de rangs inférieurs sont tenus de participer aux exercices physiques et sportifs intenses dans le cadre de leurs formations au sein de leurs unités plus fréquemment que les anciens soldats de rangs supérieurs [5]. Les formations et les stages militaires étaient depuis longtemps reconnus comme facteurs de risque des traumatismes des membres dans les forces armées [3, 9, 10]. Dans la série de Tomlinson et al. [3], 53% des traumatismes de membres étaient liées à l'entrainement et à l'exercice physique dans les unités de combat. Pour Lauder et al [5] les sports et les formations militaires étaient responsables de 33% d'hospitalisations pour des fractures de membres. Dans notre série les formations étaient les circonstances de survenue retrouvées chez 42,60% des patients.

Les fractures de stress constituent une forme particulière de fractures qui surviennent le plus souvent lors des formations et des stages militaires. Il s'agit des fractures de fatigue liées aux accidents de charge mécanique, qui résultent d'un déséquilibre ou des micro-altérations entre les processus de remodelage et de réparation osseux [11, 12]. Elles sont monnaies courantes chez les athlètes, les danseurs et les militaires recrues [13–15]. L'incidence de ces fractures de fatigue varie dans les différentes armées. Elle était en effet élevée (31%) chez les militaires israéliens de sexe masculin [16]. Dans notre étude elle était de 3,06%. Cette incidence correspond à celle rapportée dans les études américaines où elle était de 1 à 5% chez les hommes [9, 17, 18]. L'incidence élevée dans l’étude israélienne pourrait s'expliquer par le biais de recrutement des patients. En effet Milgrom C et al [16] ont étudié la survenue des fractures de stress uniquement chez les recrues qui sont généralement soumises à d'intenses activités physiques. En dehors des formations militaires, l'implication des accidents de la voie publique n'est pas négligeable. Ils occupent la deuxième place des circonstances de survenue avec une fréquence de 39,43% et sont pourvoyeurs d'un plus grand nombre de fractures (45,88%) et de lésions associées dans notre série. Cette situation n'est pas particulière aux militaires car des études réalisées au Togo par Abalo et al [19] ont montré que les accidents de la voie publique sont responsables de 57,4% de fractures de membres. Avec une fréquence de 14,31%, les accidents de sport occupaient le troisième rang des circonstances de survenues et étaient responsables de 12,78% des fractures de membres. Ces sports étaient essentiellement dominés par le ballon militaire (38,89%) qui constitue le sport collectif le plus pratiqué au sein des FDDS au Togo. Aux USA, Smith GS et al [6] ont déterminé que l´athlétisme et le sport étaient la deuxième cause des hospitalisations au sein de l'armée. Les militaires et les agents de sécurité constituent une population cible des accidents par les armes à feu. Ces accidents sont surtout fréquents en situations de conflits et de guerres [20, 21]. Dans notre série peu de fractures étaient dues aux armes à feu (3,66%) en raison de l'absence d'un contexte de conflits armés. Néanmoins les militaires et les agents de sécurité demeurent une population en danger au niveau des frontières, lors des patrouilles, et pendant les opérations de démantèlement des réseaux de cambrioleurs. La particularité de ces traumatismes de membres entrainés par les armes à feu est qu'ils s'accompagnent fréquemment de lésions multitissulaires (peau, muscle, os, vaisseaux, nerf) qui peuvent entrainer de longues périodes d'invalidité avec d'importantes perte en terme de journées de travail. Ces journées de travail perdues étaient estimées à 14009,5 jours par an chez nos patients avec une corrélation statistiquement significative à l'existence de lésions osseuses multiples et de lésions associées. Au sein de l'armée américaine, ces journées de travail perdues étaient estimées à 29435 jours par an [5].

## Conclusion

Les fractures de jambes occupent le premier rang des fractures de membres en rapport avec l'exercice de la profession militaire. Les formations-stages militaires et les accidents de la voie publique en sont les deux grandes circonstances de survenue. En raison des longues périodes d'incapacité de travail, des restrictions d'aptitudes et des invalidités que ces lésions occasionnent, des mesures préventives doivent être prises au niveau de tous les échelons de la hiérarchie militaire en vue de garantir la capacité opérationnelle des troupes.
